# Establishing Bovine Embryonic Stem Cells and Dissecting Their Self-Renewal Mechanisms

**DOI:** 10.3390/ijms26083536

**Published:** 2025-04-09

**Authors:** Ningxiao Li, Zhen Yang, Yue Su, Wei Ma, Jianglin Zhao, Xiangyan Wang, Wenjing Wan, Shengcan Xie, Heqiang Li, Ming Wang, Yiyu Zhao, Shiyao Han, Tianle Li, Shuangyi Xiehe, Jintong Guo, Linxiu Yue, Xiaoting Li, Ahui Wang, Fenfen Jiang, Suzhu Qing, Xinfeng Liu, Jun Liu, Anmin Lei, Young Tang

**Affiliations:** 1 Shaanxi Centre of Stem Cells Engineering & Technology, Key Laboratory of Livestock Biology, College of Veterinary Medicine, Northwest A&F University, Xianyang 712100, China; lningxiao@nwafu.edu.cn (N.L.); youngzhen@nwafu.edu.cn (Z.Y.); sysuyue1993@163.com (Y.S.); mawei2000@nwafu.edu.cn (W.M.); wenjingwan@nwafu.edu.cn (W.W.); xieshengcan@nwafu.edu.cn (S.X.); zhaoyiyu@nwafu.edu.cn (Y.Z.); hanshiyao@nwafu.edu.cn (S.H.); 2020010670@nwafu.edu.cn (T.L.); xhsy@nwafu.edu.cn (S.X.); 2023050645@nwafu.edu.cn (J.G.); ylx2021@nwafu.edu.cn (L.Y.); lixaoting@nwafu.edu.cn (X.L.); 2024060400@nwsuaf.edu.cn (A.W.); ffj@nwafu.edu.cn (F.J.); anmin@nwafu.edu.cn (A.L.); 2 Key Laboratory of Animal Biotechnology of the Ministry of Agriculture, College of Veterinary Medicine, Northwest A&F University, Xianyang 712100, China; zhaojianglin@nwafu.edu.cn (J.Z.); lihq@nwafu.edu.cn (H.L.); 32008057@mail.imu.edu.cn (M.W.); suzhuqing@163.com (S.Q.); 3 Key Laboratory of Ministry of Education for Conservation and Utilization of Special Biological Resources in the Western, Ningxia University, Yinchuan 750021, China; wxy805841382@126.com (X.W.); liu2019074@nxu.edu.cn (X.L.)

**Keywords:** bovine, embryonic stem cells, pluripotency, signaling

## Abstract

Bovine pluripotent stem cells (PSCs) hold significant potential for diverse applications in agriculture, reproductive biotechnology, and biomedical research. However, challenges persist in establishing stable bovine PSC lines and understanding the mechanisms underlying their pluripotency maintenance. Here, we derived bovine embryonic stem cells (bESCs) from Holstein cattle embryos. These cells exhibited robust differentiation capacity into three germ layers in vitro and in vivo. Transcriptome analysis revealed distinct molecular profiles compared to primed-state bESCs. Notably, bESC proliferation ceased on methanol-treated feeder cells, in contrast to mouse ESCs (mESCs), which proliferated normally. Pathway analysis identified key signaling events critical for bESC survival and proliferation, highlighting species-specific regulatory mechanisms. Furthermore, the derived bESCs demonstrated chimerism capacity in early bovine embryos, underscoring their functional pluripotency. This work provides a foundation for advancing bovine embryology research and stem cell-based biotechnologies in livestock.

## 1. Introduction

Pluripotent stem cells (PSCs), including embryonic stem cells (ESCs) and induced PSCs (iPSCs), possess the capacity to differentiate into all somatic cell lineages [1], making them invaluable tools for developmental studies, genomic engineering, and regenerative medicine. ESCs were first isolated from murine blastocysts [2,3] and in vitro-fertilized (IVF) human embryos [4]. Mouse ESCs (mESCs) derived from pre-implantation embryos are referred to as being in a naïve state of pluripotency [5], which includes dome-shaped colony morphology, dependency on leukemia inhibitory factor (LIF) signaling in propagation, and being more amenable to single-cell colonization and gene targeting [6,7]. In contrast, primed pluripotent human ESCs, similar to mouse epiblast stem cells derived from peri-implantation embryos with limited in vivo differentiation capacity [8,9,10], possess flat colony morphology, rely on basic fibroblast growth factor (bFGF)/Activin A signaling for self-renewal, and being sensitive to single-cell dissociation [11,12]. While murine and human ESCs have been extensively characterized, deriving stable ESC lines from domestic ungulates, such as cattle, remains a formidable challenge due to species-specific differences in signaling pathways, epigenetic regulation, and early embryonic development.

Cattle are the cornerstone of global agriculture. Establishing bona fide bESCs could revolutionize genetic improvement programs, enable precise genome editing for disease resistance or productivity traits, and serve as a model for studying pre-implantation development in large animals. Initial attempts to isolate bovine ESCs (bESCs) from the inner cell mass (ICM) of bovine embryos began over two decades ago [13]. However, extrapolation of mouse and human ESC-derivation knowledge to generate stable ESC lines from domestic ungulates and large livestock species has been challenging [14]. Recent advances have reported primed-like bESCs [15,16], which are monolayered and irresponsive to LIF in culture, bovine expanded potential stem cells (bEPSCs) with the capacity to differentiate into both embryonic and extra-embryonic lineages [17], and bovine iPSCs (biPSCs) [18,19]. Still, the signaling networks governing pluripotency in cattle remain poorly defined, and the progress in understanding bESC species specificity lags behind, with additional investigations needed to validate the pluripotency of different bovine PSCs.

In this study, we derived bESCs from IVF Holstein embryos using optimized culture conditions. These cells displayed molecular and functional hallmarks of pluripotency, including long-term genetic stability, single-cell clonogenicity, and teratoma formation. Comparative transcriptomics revealed unique regulatory pathways distinguishing our bESCs from primed-state lines, offering insights into species-specific pluripotency maintenance.

## 2. Results

### 2.1. Derivation of Bovine Embryonic Stem Cells

**Figure 1:**
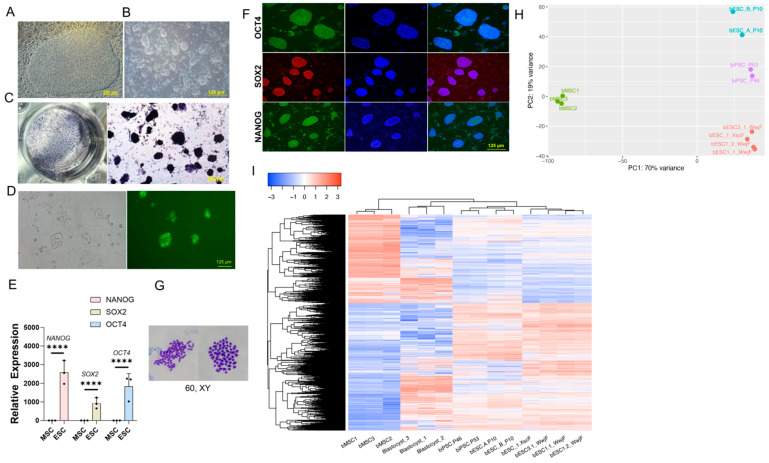
Derivation of bovine ESCs. (**A**) Colony shape initially emerging from seeded blastocysts. Bar = 250 µm. (**B**) Colony shape after passage 4. Bar = 120 µm. (**C**) Alkaline phosphatase staining of the bESC colonies. Bar = 125 µm. (**D**) SSEA4 staining of the bESCs. Bar = 125 µm. (**E**) qPCR analysis of the bESCs compared with bovine mesenchymal stem cells (bMSCs), with GAPDH as the internal control. The bar represents the mean ± “std”; ****: *p* < 0.0001; *n* = 3. (**F**) Immunostaining of the expression of pluripotent markers OCT4, SOX2, and NANOG in bESCs. Bar = 125 µm. (**G**) Karyotype analysis of bESCs. (**H**) PCA comparison of bMSCs, our derived bESCs (bESC_1, 1-1, 1-2, 3-1), biPSCs, and previously reported bESCs (bESC_A_P10, B_P10). (**I**) Heatmap of DEGs for bMSCs, bovine blastocysts, our derived bESCs (bESC_1, 1-1, 1-2, 3-1), biPSCs, and previously reported bESCs (bESC_A_P10, B_P10).

Building on our prior work establishing bovine induced pluripotent stem cells (biPSCs) [19], we optimized the culture conditions (named TIFX medium) to derive bESCs from IVF Holstein embryos sourced from the U.S. [20] and Chinese herds in this report. Colonies emerged from fresh or frozen embryos cultured on mouse embryonic fibroblast (MEF) feeders. These ESC-like cells initially emerged as relatively flat disc-like colonies (Figure 1A) and became dome-shaped following passaging (Figure 1B). Initial characterization of these cells showed strong alkaline phosphatase staining (Figure 1C) and expression of the pluripotent marker Stage-specific embryonic antigen-4 (SSEA4) (Figure 1D). These cells highly expressed endogenous pluripotency genes such as *POU5F1* (*OCT4*), *NANOG*, and *SOX2* (Figure 1E,F), exhibited a normal karyotype (Figure 1G), and were able to be passaged by single-cell colonization for more than 50 passages. RNAseq analysis was performed to compare these bESCs with the previously reported lines. Principal component analysis (PCA) of RNA-seq data revealed transcriptomic similarity to reported bovine ESCs [15] and biPSCs [19], which are distinct from somatic bovine mesenchymal stem cells (bMSCs) (Figure 1H). Differential expression analysis identified ~10,000 differentially expressed genes (DEGs) with padj < 0.05, |Log(FC)| > 1 (Table S1), between bESCs and reported bovine blastocysts [15,21], with hierarchical clustering grouping bESCs with blastocysts and the other bovine PSCs [15,19] (Figure 1I).

### 2.2. Pluripotency Characterization of Bovine Embryonic Stem Cells

**Figure 2:**
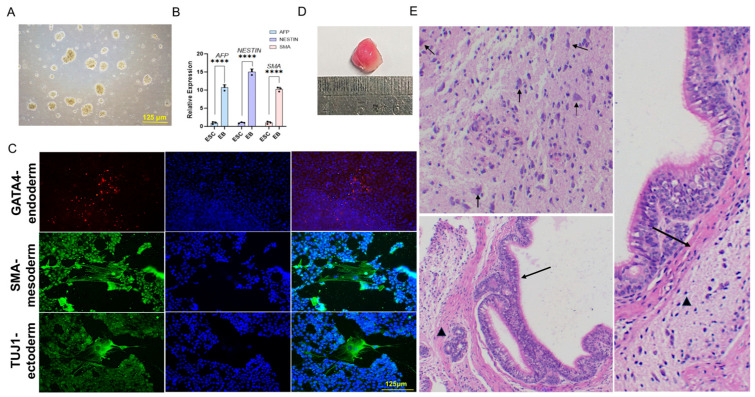
Pluripotency characterization of bESCs. (**A**) EB formed after suspension culture in serum-containing medium. Bar = 125 µm. (**B**) qRT-PCR analysis of relative expression of germ-layer-specific marker genes. *GAPDH* was used as the internal control. The bar represents the mean ± std; ****: *p* < 0.0001; *n* = 3. (**C**) Immunostaining using germ-layer-specific antibodies (anti-TUJ1 for ectoderm, anti-SMA for mesoderm, and anti-GATA4 for endoderm). Bar = 125 µm. (**D**) Teratoma formed after injection into NOD/SCID mouse. Minimal scale = 1 mm. (**E**) H&E staining showing three-germ layer differentiation of the teratoma. Top left: ectodermal pyramidal cells (arrow); bottom left: endodermal pseudostratified ciliated columnar epithelium (arrow) and tracheal gland-like structure (arrow head); right: mesodermal smooth muscle (arrow) and cartilage (arrow head).

To test the differentiation potential of the generated bESCs, an in vitro embryoid body (EB) study was performed. Upon culture in serum-containing medium in a Petri dish without pluripotency-maintaining inhibitors, these cells readily formed spherical aggregates (Figure 2A). qRT-PCR at day 5 and immunostaining at day 8 confirmed three-germ layer marker expression (Figure 2B,C). To investigate the in vivo differentiation capacity, bESCs were injected into immunodeficient NOD-SCID mice. Teratomas formed in NOD-SCID mice within 1–3 months (Figure 2D), with H&E section staining revealing ectodermal (pyramidal cells), mesodermal (smooth muscle and cartilage), and endodermal (tracheal epithelium and gland) lineages (Figure 2E). Together, the in vitro and in vivo pieces of evidence indicate the pluripotency of our bESCs.

### 2.3. The bESCs Generated Are Different in Pluripotency Status from Primed-State bESCs

**Figure 3:**
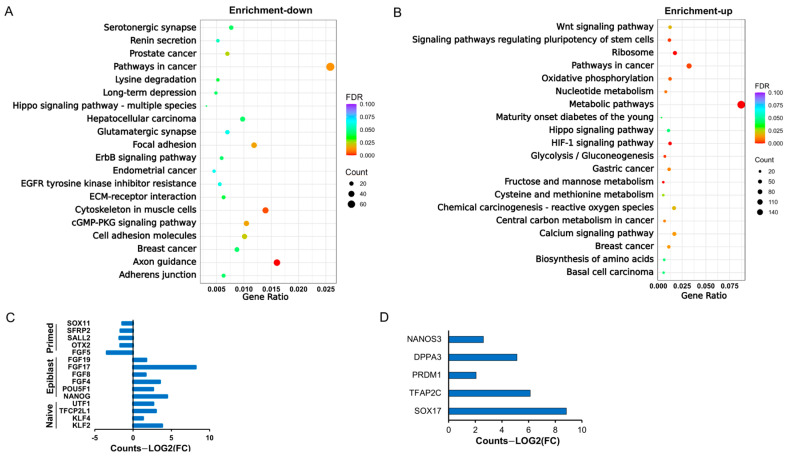
Differential signaling regulation and marker gene expression between the derived bESCs and previously reported lines. (**A**) Top 20 KEGG enrichment events from the down-regulated DGEs in our bESCs compared with reported primed-state bESCs. (**B**) Top 20 KEGG enrichment events from the up-regulated DGEs in our bESCs compared with reported primed bESCs. (**C**) Pluripotency marker expression comparison between our bESCs and reported primed bESCs based on normalized RNAseq count data, padj < 0.05. (**D**) PGC marker expression comparison between our bESCs and reported primed state bESCs based on normalized RNAseq count data, padj < 0.05.

The aforementioned RNAseq PCA revealed transcriptomic divergence between our bESCs and primed-state bESCs reported previously [15] (Figure 1H). As our bESCs form dome-shaped colonies with a clear edge and are amenable to single-cell colonization, which is more characteristic of naïve pluripotency [5] in contrast to the flat, monolayered primed pluripotent bESC colonies, we asked what cellular signaling events may differ between these two types of cells. Differential expression analysis comparing our cells versus primed-state bESCs identified 5837 DEGs (padj < 0.05, |Log(FC)| > 1) (Table S2). Of the 2648 down-regulated DEGs, Kyoto Encyclopedia of Genes and Genomes (KEGG) analysis revealed signaling events including axon guidance, serotonergic synapse, glutamatergic synapse, and cytoskeleton in muscle cells (Figure 3A, Table S3), indicating repressed neuron and muscle differentiation in our bESCs. On the other hand, of the 3190 up-regulated DEGs, enrichment signaling events included HIF-1, calcium, and Wnt signaling pathways, as well as signaling pathways regulating pluripotency of stem cells (Figure 3B, Table S3).

Also, compared with the primed bESCs, naïve pluripotency markers including *KLF2*, *-4*, *TFCP2L1*, *UTF1* [22,23], and the core pluripotent genes *OCT4* and *NANOG*, as well as key epiblast markers [23] *FGF4/8/17/19*, were all expressed at higher levels in our bESCs (Figure 3D, Table S2). On the other hand, primed pluripotency markers [23] *FGF5*, *OTX2*, *SOX11*, *SFRP2*, and *SALL2* were all expressed at greater levels in the primed bESCs (Figure 3C). Interestingly, the expressions of primordial germ cell (PGC)-specific markers, including *NANOS3*, *Stella*/*DPPA3*, *PRDM1*, *SOX17*, and *TFAP2C,* were also at greater levels in our bESCs (Figure 3D, Table S2). These data suggest a transitional stage of our bESCs between naïve and primed pluripotent states, potentially towards formative pluripotency.

### 2.4. Investigating the Species-Specific Stemness Maintenance Signaling of bESCs

**Figure 4:**
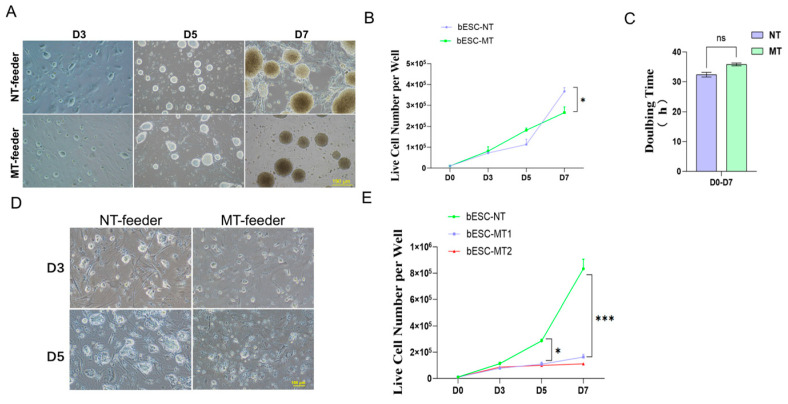
Effects of MeOH on bESC propagation. (**A**) Development of mESC colonies on regular and methanol-treated MEF feeders. Bar = 190 µm. (**B**) Counts of live mESCs grown on different feeders. The dot represents the mean ± “std”; *: *p* < 0.05; *n* = 3. (**C**) Cell doubling time of mESCs grown on different feeders. The bar represents the mean ± “std”; ns: non-significant; *n* = 3. (**D**) Development of bESC colonies on regular and methanol-treated MEF feeders. Bar = 100 µm. (**E**) Counts of live bESCs grown on different feeders after normalization based on the number of initial cells seeded. NT, MT1: 1 × 10^4^ cells/well seeded; MT2: 1 × 10^5^ cells/well seeded. The dot represents the mean ± std; *: *p* < 0.05; ***: *p* < 0.001; *n* = 3.

Our bESCs self-renew on mitomycin C-treated MEF feeders. Methanol treatment of MEF feeders was reported to support the growth of ESCs of certain species such as mice [24,25,26]. We thought to investigate the signaling specificity for bESC maintenance, by comparing the propagation of bovine and mouse ESCs on methanol-treated (MT) MEF feeders. We found that mouse ESCs (mESCs) proliferated on MT feeders similarly to how they did on non-treated (NT) control MEF feeders (Figure 4A,B), with a cell doubling time of ~33–35 h for both conditions (Figure 4C). The mESC colonies became oversized in the NT condition at day 7 with deterioration of the MEF feeders, presumably due to limited nutrients, while the integrity of MT feeders remained intact (Figure 4A). Unlike mESCs, which proliferated with similar rates on MT and CT feeders, the bESC expansion virtually ceased on MT feeders with either a regular or 10x higher number of cells seeded initially (Figure 4D,E), and with no obvious bESC colony differentiation observed. The results indicate a major deficiency in signals that promote bESC propagation when growing on MT feeders.

**Figure 5:**
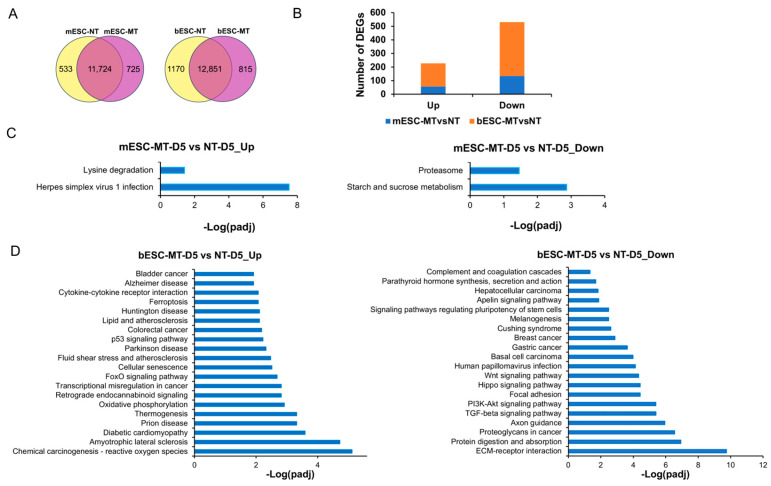
Analysis of species-specific signaling on bESC propagation. (**A**) Venn diagrams of co-expressed genes between RNAseq data on mESCs (left) and bESCs (right) grown on methanol-treated and regular feeders. (**B**) Comparison of significantly up- and down-regulated DEGs of mESCs and bESCs grown on MT versus CT feeders. (**C**) KEGG analysis of up-regulated (left) and down-regulated (right) DEGs for mESCs grown on MT versus CT feeders. (**D**) KEGG analysis of up-regulated (left) and down-regulated (right) DEGs for bESCs grown on MT versus CT feeders.

To investigate the underlying mechanisms triggering bESC propagation, comparative RNAseq analysis was performed for mouse or bovine ESCs grown on MT and CT feeders. For all detected mouse genes, only 57 and 134 DEGs (up- and down-regulated, respectively, padj < 0.05) were identified for day 5 mESCs grown in the MT versus CT condition (Figure 5A,B, Table S4). In contrast, three times more up- and down-regulated DEGs (170 and 397, respectively) were identified for day 5 bESCs grown on MT versus CT feeders (Figure 5A,B, Table S5). Compared with the CT condition, KEGG analysis of up-regulated DEGs (with *p* < 0.05 to expand the input DEG pool) of mESCs in the MT condition only showed enrichment (padj < 0.05) in herpes simplex virus 1 infection and lysine degradation (Figure 5C, left), and the down-regulated DEGs showed enrichment in starch and sucrose metabolism and proteasome (Figure 5C, right). Based on the overall observation, we considered these as cell signaling changes caused by the MT feeder condition that are not correlated with mESC self-renewal.

To identify signaling events specific for bESC propagation, KEGG analysis was performed for bESCs grown in the MT versus CT condition, and further compared with that of mESCs to remove the same enrichment terms. Different from the mESC data, KEGG analysis of the up-regulated DEGs in bESCs in the MT versus CT condition showed that they were enriched with cell death and stress-related events (padj < 0.05), including ferroptosis, cell senescence, p53 and FOXO signaling pathways, reactive oxygen species (ROS), and oxidative phosphorylation, as well as a number of neural degenerative diseases, including Alzheimer’s, Huntingdon’s, Parkinson’s, and prion diseases and amyotrophic lateral sclerosis (Figure 5D, left). On the other hand, for the down-regulated DEGs in the MT versus CT condition, enrichment events included extracellular matrix (ECM)–receptor interaction and TGF-beta, Hippo, Wnt, and Apelin signaling pathways, as well as signaling pathways regulating pluripotency of stem cells (Figure 5D, right). These data thus indicate that, unlike mESCs, cellular stress/death signaling events were up-regulated in bESCs grown in the MT condition, while the signaling pathways regulating cell survival were down-regulated, resulting in a cessation of bESC self-renewal.

### 2.5. bESCs Exhibit the Capacity to Integrate into Bovine Embryos

**Figure 6:**
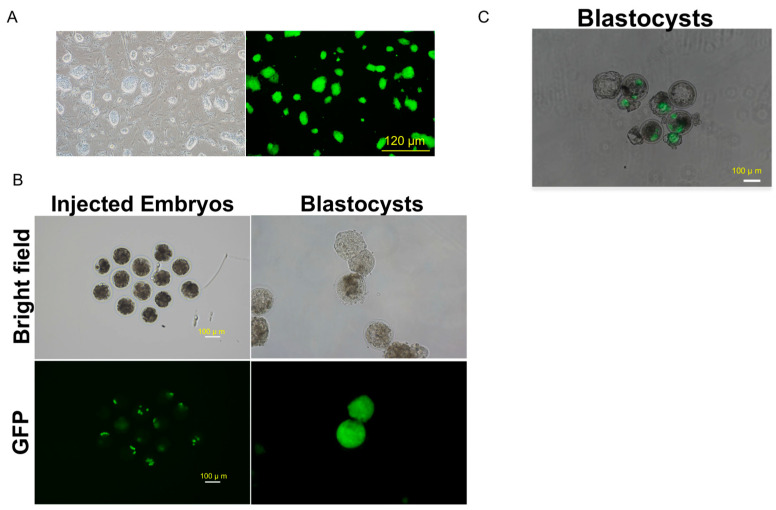
bESC chimerism test in bovine early embryos. (**A**) Representative images of the GFP-positive bESC line. Bar = 120 µm. (**B**) Images of injected embryos at 8- and 16-cell stages and developed blastocysts. Bar = 100 µm. (**C**) Overlay images of GFP-positive blastocysts. Bar = 100 µm.

To further explore the pluripotency of our bESCs, we investigated their capacity for chimerism using bovine embryos. A GFP-labeled bESC line was established (Figure 6A). IVF bovine embryos at the 8- or 16-cell stage (day 3 or 4) were injected with 8–10 GFP-positive bESCs, and then the injected embryos were left to continue to develop. After 4–5 days of culture (day 8), most injected embryos (21/26) developed into blastocysts, while some chimeric blastocysts exhibited obvious integration of GFP-positive cells in the embryos (Figure 6B,C, Table 1). These data thus indicate that the bESCs we developed possess the capacity to integrate into early bovine embryos.

## 3. Discussion

Bovine PSCs, including bESCs and biPSCs, may serve as great tools for agricultural biotechnology research, including as cell sources for gene editing for transgenic cattle generation and for cultivated meat production. Relevant findings identifying the transcription factors involved in the regulation of pluripotency and self-renewal in ESCs may provide keys that enable the derivation of ESCs in domestic species [27]. Here, we established bESCs capable of continuous self-renewal and characterized their pluripotency using a variety of in vitro and in vivo tests.

The stable propagation of large ungulate PSCs has been a challenge across the field. We dissected key signaling events and identified potential signaling pathways that are necessary for bovine ESC proliferation. The reliance of bESCs on feeder-derived signals underscores the importance of species-specific culture conditions. Unlike mESCs, which thrive on methanol-treated feeders, our bESCs arrest proliferation under these conditions, likely due to disrupted ECM interactions and activation of stress response pathways (e.g., p53, FOXO). These findings align with prior reports highlighting the sensitivity of bovine PSCs to feeder cell modifications [28], suggesting that ECM–receptor signaling and TGF-β or other pathways are indispensable for bESC self-renewal. Future studies should explore feeder-free culture systems or synthetic matrices tailored to bovine signaling requirements to enhance scalability and experimental reproducibility.

The chimerism capacity of bESCs in early bovine embryos opens new avenues for agricultural biotechnology. By integrating into host embryos, these cells could serve as vectors for introducing genetic modifications directly into the germline, bypassing the inefficiencies of somatic cell nuclear transfer. This approach holds promise for accelerating the production of transgenic cattle with enhanced disease resistance, milk yield, or climate adaptability. Moreover, bESC-derived chimeras could provide a platform to study early lineage commitment and trophoblast development in cattle, addressing gaps in our understanding of ungulate embryology.

Our transcriptomic comparisons between bESCs and primed-state lines revealed up-regulated naïve-associated markers (e.g., *KLF2/4*, *TFCP2L1*) and repressed neuronal differentiation pathways. This molecular profile suggests that bovine pluripotency may occupy a unique niche between murine naïve and primed states, necessitating revised criteria for classifying livestock PSCs. The co-expression of primordial germ cell (PGC) markers (e.g., *NANOS3*, *DPPA3*) further implies that bESCs retain a developmental plasticity akin to early epiblast cells, which could be harnessed for in vitro gametogenesis or interspecies chimerism studies. Despite these advances, challenges remain. The genetic stability of bESCs over extended passages, the potential epigenetic drift, and the need for low-cost serum-free culture systems warrant further investigation. Further verification and validation of the pluripotency state and specific signaling networks governing bESC self-renewal through qRT-PCR, protein-level, and functional analyses are warranted. Additionally, the functional contribution of bESCs to adult tissues in chimeras must be validated to confirm their pluripotent potential.

In conclusion, this study establishes a robust framework for deriving and characterizing bESCs, providing critical insights into bovine species-specific pluripotency regulation.

## 4. Materials and Methods

### 4.1. Bovine Embryo Production and Culture

Bovine ovaries were collected from a local slaughterhouse and immediately (within 2 h) transported to the laboratory. Ovaries were obtained and placed in a 100 mm culture dish (Corning, 430167, Pudong, Shanghai, China), which was prefilled with approximately 3 mL of M199 (Thermo Scientific, 11150059, Grand Island, NY, USA). Using a scalpel, the follicles on the bovine ovaries were carefully incised within the 100 mm culture dish, allowing the cumulus oocyte complexes (COCs) to flow out with the follicular fluid, enabling the collection of COCs. The collected COCs were cultured in BO-IVM medium (IVF Bioscience, Falmouth, Cornwall, UK) with 20 ng/mL LIF (Gibco, 300-05-1, Grand Island, NY, USA), 40 ng/mL EGF (R&D, 236-EG, Minneapolis, MN, USA), and 20 ng/mL IGF-1 (R&D, 291-G1) covered with mineral oil (Sigma, M5310, Burlington, MA, USA) in 4-well dishes (Thermo Scientific, 144444) for 22.5 h in a humidity incubator (Thermo Scientific, 3311) at 38.5 °C with 5% CO_2_.

At 22.5 h post-maturation, COCs were transferred to BO-IVF medium (IVF Bioscience) in 4-well dishes covered with mineral oil and incubated at 38.5 °C under 5.5% CO₂. Frozen bovine semen donated by the Shaanxi Dairy Cattle Center was used for in vitro fertilization (IVF). The straws were thawed in a 38.5 °C water bath, washed twice in BO-SemenPrep medium (IVF Bioscience) by centrifugation at 1500 rpm for 10 min, and co-cultured with 1–2 × 10^6^/mL of sperm and COCs in BO-IVF medium (IVF Bioscience). Fertilization was performed at 38.5 °C under 5.5% CO₂ for 20 h (day 0). IVF embryos were removed from the cumulus cells in hyaluronidase (Sigma, H3506) dissolved in DPBS and cultured for 8 days in BO-IVC medium (IVF Bioscience) with 0.1 μM Melatonin (MCE, HY-B0075, Shanghai, China), 20 ng/mL LIF, 40 ng/mL EGF, and 20 ng/mL IGF-1 in a humidity incubator (Thermo Scientific, 3131) at 38.5 °C with 5% CO_2_ and 5% O_2_. Embryos that developed in the blastosphere stage were used for bovine ES cell (bESC) derivation.

### 4.2. Derivation and Culture of bESCs

Bovine blastocysts produced by IVF (day 8) were used for bovine embryonic stem cell (bESC) derivation. Unhatched D8 IVF embryos were treated with 2 mg/mL Pronase (Merck Millipore, 10165921001, Grand Island, NY, USA) at 38.5 °C with 5% O_2_ and 5% CO_2_ for 1 min 45 s to remove the zona pellucida (ZP), followed by three washes in IVC medium to eliminate enzyme traces. Hatched embryos and zona pellucida-free embryos were placed in a 24-well plate containing mitomycin C-treated mouse embryonic fibroblast (MEF) feeders and cultured in TiFX medium. The TiFX medium comprised mTeSR-plus media (mTeSR Plus (STEMCELL, 100-0276, Cambridge, MA, USA), 1× Penicillin-Streptomycin (Gibco, 15140122), 0.8 μM PD184352 (MEK1/2i; Selleck Chemicals, S1020, Houston, TX, USA), 2.5 μM IWR-1 (Selleck Chemicals, S7086), 3.3 μM EPZ004777 (DOT1Li; Selleck Chemicals, S7565), 2 μM SU5402, (Selleck Chemicals, S7667), 3 μM CHIR99021 (GSK3i; Selleck Chemicals, S2924), 10 μM Forskolin (Selleck Chemicals, S2449), and 1000 U/mL human LIF (R&D Systems, 7734LF025), with the presence of 10 μM ROCK inhibitor (ROCKi) (Selleck Chemicals, S1049). Cultures were maintained at 37 °C under 5% CO₂ for 48 h without disturbance. After 7–8 days, embryonic outgrowths were dissociated using TrypLE Express (Gibco, 12604039) and passaged in a 12-well plate with feeder cells in the presence of 10 μM ROCKi. Distinct dome-shaped colonies began to emerge from passage 1 (P1) to passage 3 (P3) and were replated onto fresh feeder cells. The established bESC line was passaged every three days at a 1:5 ratio using TrypLE Express in TiFX medium containing 10 μM ROCKi.

### 4.3. Quantitative Reverse Transcription PCR (qRT-PCR)

To isolate total RNA, the miRNeasy Micro Kit (Qiagen, 217084, Stockach, Germany) was utilized, followed by the removal of genomic DNA through incubation with DNase I (Qiagen, 1011132). Subsequently, 0.5 mg of RNA was reverse-transcribed into cDNA using PrimeSTAR^®^ Max DNA Polymerase (Takara, R045B, San Jose, CA, USA). Quantitative reverse transcription PCR (qRT-PCR) was conducted using ChamQ SYBR qPCR Master Mix (Vazyme, Q311-02AA, Nanjing, China) on the QuantStudio™ 1 system (Applied Biosystems, Foster City, CA, USA). For normalization of gene expression, GAPDH or β-Actin served as the housekeeping gene. The relative expression levels of target genes were calculated using the ΔΔCt method, with β-Actin or GAPDH as the reference, and analyzed using the software provided with the QuantStudio™ 1 platform.

### 4.4. Embryoid Body Differentiation

EB formation experiments were carried out with bESC lines. When growing to 70–80% confluency with mainly middle-size colonies, the cells were treated with freshly prepared 1 mg/mL collagenase for 30 min and removed from the plate by pipetting. After three washes with DMEM/F12, the cells were plated onto low-adhesive Petri dishes in EB formation medium (1:1 ratio of TiFX and DMEM, supplemented with 10% KSR (Gibco, 10828028)). Half of the medium was changed to DMEM with 10% FBS every other day. EBs were treated with 0.05% Trypsin (Gibco, 15400054) 5 days later and plated onto gelatin (Merck, ES-006-B)-coated 12-well plates (Corning, 3336). The cells were subjected to immunofluorescence staining after another 7 days of culture.

### 4.5. Immunostaining

The cells underwent an initial fixation in 4% paraformaldehyde (PFA) (Solarbio, P110, Beijing, China) for 15 min at ambient temperature, followed by treatment with 0.5% Triton X-100 in PBS for 15 min to permeabilize the membranes. Subsequently, the cells were blocked with 5% goat serum (Beyotime, C0265, Shanghai, China) overnight. They were then incubated in a goat serum solution containing primary antibodies for 2 h at 37 °C, and afterward with secondary antibodies at room temperature for 1 h. Primary antibodies, including OCT4 (Bioss, BS-0830R, Woburn, MA, USA), SOX2 (Bioss, BS-0523R), NANOG (Bioss, BS-10408R), GATA4 (Invitrogen, YB370493, Waltham, MA, USA), TUJ1 (Invitrogen, MA1-118), and SMA (Invitrogen, MA1-06110), were diluted according to the manufacturers’ instructions. Alexa Fluor 488- or 594-conjugated donkey anti-rabbit or donkey anti-mouse secondary antibodies (Proteintech, SA00003-8, SA00014-4, Rosemont, IL, USA) were utilized at a 1:1000 dilution. Cells were counterstained with DAPI and mounted on slides using Fluoroshield (Merck, F6057). Images of the slides were captured using the EVOS M7000 Cell Imaging System (Invitrogen).

### 4.6. Teratoma Assay

Before TrypLE digestion, bESCs were treated with 10 μM ROCK inhibitor (ROCKi) for 2 h. After digestion and centrifugation, the cells were resuspended in a solution containing 30% cold Matrigel (Corning, 354230) in DMEM/F12 medium and 10 μM ROCKi. A total of 5 × 10^6^ bESCs were injected into the hind legs of 10-week-old male NOD-SCID mice (406 NODSCID, Charles River, Beijing, China) at 100 μL per injection. Six to eight weeks later, the teratomas were dissected and fixed in 4% paraformaldehyde (PFA). The fixed teratomas were paraffin-embedded, sectioned, and stained with hematoxylin and eosin for histological examination.

### 4.7. RNAseq Library Preparation and Sequencing

Total RNA was isolated using the miRNeasy Mini Kit (Qiagen, 217004). The integrity of the RNA was evaluated using the RNA Nano 6000 Assay Kit on the Bioanalyzer 2100 system (Agilent Technologies, Santa Clara, CA, USA). Total RNA served as the input material for RNA sample preparations. Fragmentation was executed using divalent cations at elevated temperatures in First-Strand Synthesis Reaction Buffer (5X). First-strand cDNA synthesis was achieved utilizing random hexamer primers and M-MuLV Reverse Transcriptase (RNase H-). Subsequent second-strand cDNA synthesis was conducted using DNA Polymerase I and RNase H. The library fragments were purified using the AMPure XP system. PCR amplification was performed with Phusion High-Fidelity DNA polymerase, universal PCR primers, and an index (X) primer. Final PCR products were purified with the AMPure XP system, and library quality was assessed on the Agilent Bioanalyzer 2100 system. Sequencing was executed on an Illumina NovaSeq 6000 (v1.5 chemistry) with paired-end 100 bp reads (50 million reads/sample) by Novogene (Tianjing City, China).

### 4.8. Transcriptome Data Analysis

Raw data (raw reads) in fastq format were initially processed using fastp software v0.23.2. Reference genome and gene model annotation files were obtained directly from genome websites. The reference genome index was constructed using Hisat2 v2.0.5, and paired-end clean reads were aligned to the reference genome using Hisat2 v2.0.5. FeatureCounts v1.5.0-p3 was employed to enumerate the reads mapped to each gene. The FPKM (fragments per kilobase of transcript per million mapped reads) of each gene was computed based on gene length and mapped read counts. Further RNAseq data analysis was conducted on the NovoMagic platform (https://magic-plus.novogene.com, accessed on 1 December 2024). Differential expression analysis between two conditions/groups (two biological replicates per condition) was performed using the DESeq2 R package (1.20.0). Additionally, differential expression analysis was conducted using the edgeR R package (3.22.5). Gene Ontology (GO) enrichment and KEGG pathway analysis of differentially expressed genes were implemented via the clusterProfiler R package, which corrects for gene length bias.

Additional RNA-seq data analysis was conducted on usegalaxy.org. Sequencing adapters and low-quality reads were trimmed using Cutadapt before mapping. Post-filtering read quality was evaluated using fastQC. For mapping, bovine genomic sequences and RefSeq gene coordinates (ARS-UCD1.2/bosTau9) were downloaded from the UCSC genome browser. All filtered reads were aligned to the bovine reference genome using RNA STAR (Galaxy Version 2.7.8a) with default parameters. Read counts per gene were quantified using featureCounts (Galaxy Version 2.0.1). Differentially expressed genes between samples were identified using DESeq2 (Galaxy Version 2.11.40.6+galaxy1) with default parameters, producing a principal component analysis plot and a heatmap of the sample-to-sample distance matrix. The most differentially expressed genes (adjusted *p*-value < 0.05) were extracted from the DESeq2 results with an absolute fold change (FC) >5. Normalized counts for these differentially expressed genes and their Z-scores were calculated on the Galaxy platform and visualized as heatmaps using Heatmap2 (Galaxy Version 3.0.1). Normalized counts were subjected to DAVID and integrated KEGG analysis (https://davidbioinformatics.nih.gov/tools.jsp, accessed on 1 December 2024).

### 4.9. Karyotyping

To perform karyotyping on various bESC lines, the cells were initially exposed to 10 μg/mL Demecolcine (MCE, HY-N0282) for 3 h at 37 °C. After this treatment, the cells were collected through trypsinization. Subsequently, the cells were subjected to a hypotonic solution (0.56% KCl) and incubated for 15 min at 37 °C. Following this, the cells were washed three times with a fixative solution composed of methanol and glacial acetic acid in a 3:1 ratio. The fixed cells were then carefully dropped onto pre-chilled, wet glass slides. Once the slides were dry, they were stained with Giemsa solution (Beyotime, C0133) diluted at a ratio of 1:20. Finally, the nuclei were examined using a Nikon microscope equipped with a 100× oil immersion objective lens.

### 4.10. bESC Proliferation Assay on Methanol-Treated MEF Feeders

MEF cells were resuspended and seeded into each well of gelatin-coated 12-well plates in DMEM with 10% FBS, to achieve a final plating density of 1 × 10^5^ cells/well. Cells were treated with pure methanol for 5 min and then rinsed with DPBS three times. Then, 1 × 10^5^ mESCs or 1 × 10^5^–1 × 10^6^ bESCs were seeded into each well of gelatin-coated 12-well plates. mESCs were cultured in “2i/LIF” medium [5] (1:1 DMEM/F12 and Neurobasal media, with 50× N2 (Gibco, N2A1370701), 100× B27 (Gibco, 17504044), 20% KSR (Gibco, 10828028), 1 mM L-glutamine, 0.1 mM non-essential amino acids, 50 µM β-mercaptoethanol, 1000 U/mL mouse leukemia inhibitory factor (mLIF, Gibco A35933), 1 µM PD0325901(MCE, HY-131295), and 3 µM CHIR99021), and bESCs were cultured in TiFX media with 10 μM ROCKi. Cells were grown on methanol-treated or non-treated MEF feeder cells at 37 °C in a humidified incubator. Live cells were counted every two days until day 7.

### 4.11. In Vivo Bovine Chimera Assay

The bovine embryos used for the chimera assay were prepared following the IVM and IVF procedures described in the aforementioned Bovine Embryo Production and Culture protocol. GFP-labeled bovine embryonic stem cells (bESCs), generated via piggyBac transposition, were microinjected into bovine embryos using a micromanipulator (Eppendorf, TransferMan^®^ 4r) to create chimeras. Day 3 (8-cell) and day 4 (16-cell) embryos were microinjected with 6-8 GFP-labeled bESCs in IVC media. The microinjected chimera embryos were then cultured in IVC media in a humidity incubator at 38.5 °C with 5% CO_2_ and 5% O_2_ until day 8. On day 8, the embryos were examined under a fluorescence microscope (Nikon, Ti2-U) to assess the chimerism rate based on GFP expression.

### 4.12. Statistical Analysis

One-way ANOVA with Tukey’s multiple comparison test or Student’s *t*-test was used for data analysis. The figures are presented as the mean ± standard deviation (std). A *p*-value < 0.05 (*) was considered statistically significant. All data were analyzed with the SPSS platform v29.0.

## Figures and Tables

**Table 1:** Summary of bESC–bovine embryo chimerism experiments.

Experiment	Initially Injected Embryos	Blastocysts Developed	Chimeric Blastocysts	Chimeric Blastocyst Ratio
1	13	9	4	44%
2	13	13	6	46%

## Data Availability

The original data presented in the study is available in NCBI Geo Datasets (GSE290793).

## Supplementary Materials

The following supporting information can be downloaded at https://www.mdpi.com/article/10.3390/ijms26083536/s1.
