# Supplementary material for: Establishing Bovine Embryonic Stem Cells and Dissecting Their Self-Renewal Mechanisms
Source: Int J Mol Sci. 2025 Apr 9;26(8):3536. doi: 10.3390/ijms26083536 (PMC12027403; doi:10.3390/ijms26083536)
Supplement: Supplementary file 1 [file ijms-26-03536-s001.zip › Supplementary Files 2.pdf]

Establishing Bovine Embryonic Stem Cells and Dissecting Their Self-renewal Mechanisms

Ningxiao Li 1# , Zhen Yang 1# , Yue Su 1# , Wei Ma 1† , Jianglin Zhao 2† , Xiangyan Wang 3† , Wenjing Wan 1† , Shengcan Xie 1† , Heqiang Li2 , Ming Wang 2 , Yiyu Zhao 1 , Shiyao Han 1 , Tianle Li 1 , Shuangyi Xiehe 1 , Jintong Guo 1 , Linxiu 5, Yue 1 , Xiaoting Li 1 , Ahui Wang 1 , Fenfen Jiang1, Suzhu Qing 2 , Xinfeng Liu 3*, Jun Liu 2*, Anmin Lei 1*, and Young Tang 1,3,

Shaanxi Centre of Stem Cells Engineering & Technology, Key Laboratory of Livestock Biology, Engineering 7
Research Center of Efficient New Vaccines for Animals, Ministry of Education and Universities of Shaanxi 8
Province, College of Veterinary Medicine, Northwest A&F University, Yangling, Shaanxi, China

[2]
College of Veterinary Medicine, Northwest A&F University, Key Laboratory of Animal Biotechnology of the Ministry of Agriculture, Yangling, Shaanxi, 712100, People's Republic of China

[3]
Key Laboratory of Ministry of Education for Conservation and Utilization of Special Biological Resources in 14
the Western, Ningxia University, Yinchuan, Ningxia, 750021, China

Supplementary Tables

Table S1-Normalized counts for bovine ESCs, MSCs and embryos

Table S2-DEGs for bESCs vs primed-bESCs

Table S3-KEGG Enrichment for bESCs vs Primed-bESCs

Table S4-DEGs for mESC MT- vs NT-condition

Table S5-DEGs for bESC MT- vs NT-condition
